# Effect of omega-3 polyunsaturated fatty acid on endometriosis

**DOI:** 10.1016/j.clinsp.2025.100654

**Published:** 2025-04-23

**Authors:** Erniao Liu, Qianting Wang, Yaoxian Bai, Xi Zhang, Jingfang Wang

**Affiliations:** a Department of Gynaecology and Obstetrics, The Second Hospital of Shanxi Medical University, Shanxi, China; b Department of Family Planning Surgery, Taiyuan Maternity and Child health Care Hospital, Taiyuan, China; c Department of Gynaecology and Obstetrics, GaoPing People's Hospital, Gaoping, China

**Keywords:** Omega-3 polyunsaturated fatty acids, Endometriosis, Meta-analysis

## Abstract

**Objective:**

This study aimed to evaluate the effect of Omega-3 Fatty Acids (ω-3 PUFAs) on endometriosis.

**Design:**

The authors conducted a comprehensive search of the PubMed, Embase, Cochrane Library and Web of Science databases, focusing exclusively on Randomised Controlled Trials (RCTs) to study the impact of ω-3 PUFAs on endometriosis. The included studies were assessed for methodological quality using the Cochrane bias risk assessment tool and analyzed using data analysis software.

**Results:**

The search yielded five RCTs conducted between the database's inception and July 2023, with a total sample size of 424 patients with endometriosis. The meta-analysis results showed no statistically significant effects of ω-3 Polyunsaturated Fatty Acids (PUFAs) on pain (Mean Difference [MD = -0.387], 95 % Confidence Interval [95 % CI -1.742–0.967], *I*^2^ = 93.3 %, *z* = 0.56, *p* = 0.575), sexual activity (MD = 0.143, 95 % CI -0.210–0.497, *I*^2^ = 0 %, *z* = 0.79, *p* = 0.427), pain intervention (MD = -0.216, 95 % CI -0.717–0.285, *I*^2^ = 0.0 %, *z* = 0.84, *p* = 0.399), catastrophic thinking (MD = 0.158, 95 % CI -0.315–0.632, *I*^2^ = 0.0 %, *z* = 0.66, *p* = 0.512) and the 12-item short form health survey (MD = 0.001, 95 % CI -0.053–0.503, *I*^2^ = 0.0 %, *z* = 0.00, *p* = 1.000), which were all statistically insignificant. However, ω-3 PUFAs appeared to reduce the inflammatory response in patients with endometriosis (MD = -5.20, 95 % CI -6.21–-4.20, *I*^2^ = 0 %, *z* = 10.13, *p* < 0.001).

**Conclusion:**

Based on the available evidence, ω-3 PUFAs may reduce the inflammatory response in patients with endometriosis, specifically by decreasing levels of pro-inflammatory cytokines, such as TNF-alpha, IL-6 and IL-1, indicating potential anti-inflammatory properties that warrant further investigation.

**Trial registration:**

PROSPERO: CRD42023441699.

## Highlights

• ω−3 PUFAs had no significant effect on endometriosis symptoms, such as pain, etc.
• ω−3 PUFAs could reduce the inflammatory response in patients with endometriosis.
• The local environment of implants exhibits pre-inflammatory tissue characteristics.

## Introduction

Endometriosis is a chronic, estrogen-dependent condition affecting women of childbearing age. It is defined by the presence of endometriosis-like lesions, consisting of endometrial stroma, glandules, or tissue outside the uterine cavity, grows outside the uterine cavity. This misplaced tissue can adhere to various pelvic organs, such as the ovaries, fallopian tubes, and the peritoneum, often leading to pain, inflammation, and in some cases, infertility.^1^, ^2^, ^3^ Although progressive, endometriosis is frequently diagnosed 7–10 years after symptom onset. The predominant symptoms include chronic abdominal pain, dysmenorrhoea, and deep-seated pain. Additionally, endometriosis can impair fertility in women of childbearing age, with prevalence rates ranging from 3 % to 33 %.^4^ The incidence of pelvic pain and/or infertility in women varies between 30 % and 50 %.^5^ To date, there are limited medical treatments that substantially reduce or eradicate endometriosis.^6^ The most widely accepted clinical approach is pharmacological hormone therapy, designed to lower circulating estrogen levels.^7^ Unfortunately, long-term hormone use can cause severe side effects, such as osteoporosis and symptoms of premature menopause, thus failing to provide the desired outcomes. Consequently, there is an urgent need for new treatment strategies with minimal side effects that effectively alleviate lesions and symptoms in patients. Given the limitations of hormonal therapies, attention has turned to dietary interventions targeting inflammatory pathways. One potential strategy involves the use of Polyunsaturated Fatty Acids (PUFAs), particularly omega-3 (ω−3) subtypes, which are known for their anti-inflammatory properties.^8^

PUFAs have shown promising anti-proliferative, anti-inflammatory and anti-apoptotic effects.^9^ Research suggests that women with endometriosis may lack PUFAs in their uterine microenvironment, potentially impairing endometrial function. Among PUFAs, ω−3 subtypes have garnered particular interest due to their distinct biological roles. Specifically, ω−3 PUFAs, including Eicosapentaenoic Acid (EPA) and Docosahexaenoic Acid (DHA), are primarily found in marine organisms and their derivatives.^10^ Unlike pro-inflammatory ω−6 PUFAs, ω−3 subtypes compete for enzymatic pathways, thereby modulating prostaglandin synthesis and inflammatory responses.^11^, ^12^, ^13^ In particular, EPA and DHA are effective in modulating the immune response by suppressing genes involved in inflammation and by substituting ω−6 PUFA and cholesterol to modify the cell membrane composition, thus reducing inflammation and pain.^14^ An in vitro study reported that the survival of endometrial cells in women with and without endometriosis is influenced by the fatty acid content in the culture medium.^15^ Additionally, ω−3 PUFAs may prevent the development of endometriosis.^4^ A prospective cohort study by Missmer et al. demonstrated that long-term consumption of ω−3 PUFAs substantially reduces the risk of endometriosis.^16^ Proctor also mentioned in an article that the intake of ω−3 PUFA (fish oil) can alleviate the pain and inflammation associated with endometriosis.^17^ In typical endometriosis lesions, excessive estrogen promotes the production of large quantities of prostaglandins, inducing inflammation and pain.^18^ While ω−3 has been established to have anti-inflammatory properties^19^ by selectively regulating specific prostaglandins, whereas vitamin B6 aids in the production of oestradiol prostaglandins.^20^

Despite extensive research, the relationship between PUFAs and endometriosis remains controversial. Variations in study design, sample size, and methodology contribute to inconsistent findings, underscoring the need for a comprehensive systematic review and meta-analysis. Therefore, this study aims to evaluate the therapeutic impact of ω−3 PUFAs on endometriosis symptoms and inflammatory markers through a systematic review and meta-analysis, thereby providing evidence-based recommendations for clinical practice.

## Materials and methods

### Search strategy

The researchers searched four electronic databases (Pubmed, EMBASE, Cochrane Central Register of Controlled Trials and Web of Science) between inception and July 2023. The search strategy was based on the PICOS framework: (P) Population: patients with endometriosis; (I) Intervention: administration of ω−3 PUFA formulations; (C) Control group: participants receiving placebo or olive oil; (O) Outcomes: pain, improvement in sexual activity, cytokines, catastrophic thinking and results from the 12-item Short-Form health survey (SF-12); (S) Study design: Randomised Controlled Trials (RCTs). The detailed search string used for PubMed was as follows (Table 1):

1. "Endometriosis"[MeSH];
2. ((((Endometriosis[Title/Abstract]) OR Endometrioses[Title/Abstract]) OR Endometrioma[Title/Abstract]) OR Endometriomas[Title/Abstract]);
3. Combined #1 OR #2 to include both MeSH terms and Title/Abstract terms for endometriosis;
4. "Fatty Acids ω−3″[MeSH];
5. (((((((Fatty Acids ω−3[Title/Abstract]) OR ω−3 Fatty Acid[Title/Abstract]) OR Acid ω−3 Fatty[Title/Abstract]) OR Fatty Acid ω−3[Title/Abstract]) OR ω−3 Fatty Acid[Title/Abstract]) OR ω−3 Fatty Acids (ω−3 PUFAs) [Title/Abstract]) OR n-3 Oil[Title/Abstract]) OR Oil n-3[Title/Abstract]) OR n-3 Oil[Title/Abstract]) OR n3 Oil[Title/Abstract]) OR Oil n3[Title/Abstract]) OR n-3 Fatty Acids[Title/Abstract]) OR Oil,Oil n-3,n 3 Oil,n3 Oil,Oil n3,n-3 Fatty Acids,[Title/Abstract]) OR n-3 Fatty Acids [Title/Abstract]) OR ω−3 Fatty Acids[Title/Abstract]) OR n-3 PUFA [Title/Abstract]) OR PUFA n-3 [Title/Abstract]) OR n-3 PUFA;
6. Combined #4 OR #5 to include both MeSH terms and Title/Abstract terms for ω−3 PUFAs;
7. Applied filter for randomized controlled trials: randomized controlled trials[Publication Type];
8. Final search query: #3 AND #6 AND #7.

**Table 1:** Search strategies on PubMed.

#1	"Endometriosis"[MeSH]
#2	(((((Endometriosis[Title/Abstract])OR Endometrioses[Title/Abstract]) OREndometrioma[Title/Abstract]) OR Endometriomas[Title/Abstract]
#3	#1 OR #2
#4	"Fatty Acids, Omega-3″[MeSH]
#5	(((((((Fatty Acids, Omega-3[Title/Abstract]) OR Omega-3 Fatty Acid[Title/Abstract]) OR Acid, Omega-3 Fatty[Title/Abstract]) OR Fatty Acid, Omega-3[Title/Abstract]) OR Omega 3 Fatty Acid[Title/Abstract]) OR Omega-3 Fatty Acids[Title/Abstract]) OR n-3 Oil[Title/Abstract]) OR Oil, n-3[Title/Abstract]OR n 3 Oil[Title/Abstract]) OR n3 Oil[Title/Abstract]) OR Oil, n3[Title/Abstract]) OR n-3 Fatty Acids[Title/Abstract]) OR Oil,Oil, n-3,n 3 Oil,n3 Oil,Oil, n3,n-3 Fatty Acids,[Title/Abstract]) OR n 3 Fatty Acids [Title/Abstract]) OR Omega 3 Fatty Acids[Title/Abstract]) OR n-3 PUFA [Title/Abstract]) OR PUFA, n-3 [Title/Abstract]) OR n 3 PUFA [Title/Abstract]) OR n3 Fatty Acid [Title/Abstract]) OR Fatty Acid [Title/Abstract]) OR n3[Title/Abstract]) OR n3 PUFA[Title/Abstract]
#6	#4 OR #5
#7	randomzied controlled trials[Publication Type]
#8	#3 AND #6 AND #7

### Selection criteria

The inclusion criteria were as follows: 1) Studies where the experimental group received ω−3 PUFA supplements as an intervention for endometriosis; 2) The control group received placebo or olive oil; 3) Clinical RCTs; and 4) Outcomes measured included at least one of the following: pain, improvement in sexual activity, cytokines, catastrophic thinking, and the SF-12 survey.

### Exclusion criteria

Studies were excluded if they (1) had incomplete or unreported data or (2) were not RCTs, including meeting summaries, case reports, or letters.

### Risk of bias in individual studies

Two researchers independently assessed the risk of bias using the Cochrane Handbook version 5.1.0 tool (http://www.cochrane.org/handbook, London, UK) for evaluating risk in RCTs. They considered seven domains: 1) Random sequence generation, 2) Concealment of treatment allocation, 3) Blinding of participants and personnel, 4) Completeness of outcome data, 5) Selective reporting and 6) Other potential sources of bias. Studies were categorized based on the number of high-risk components as having a high risk (five or more), medium risk (three or four) or low risk (two or fewer) of bias.^21^

### Data analysis

In the research conducted, all variables were continuous and expressed as standard deviations.^22^ The continuous variables were reported as mean difference (MD = the absolute difference between the mean values of the treatment and control groups, calculated using the same scale) or standardized MD (SMD = the MD between groups divided by the standard deviation across participants, used to combine data from experiments with different scales), including a 95 % Confidence Interval (95 % CI) for analysis. Heterogeneity among the results was statistically assessed using *Q*-tests and the *I*^2^ statistic.^23^ A p-value of > 0.05 in the *Q*-test suggests low heterogeneity, whereas a p-value of 〈 0.05 indicates significant heterogeneity. The *I*^2^ value quantifies the percentage of variation across studies due to heterogeneity rather than chance. Its values of 25 %, 50 % and 75 % indicate low, medium and high heterogeneity, respectively. A fixed-effects model was used if *I*² ≤ 50 % and p 〉 0.1; otherwise, a random-effects model was applied. Subgroup analysis (e.g., by dosage, intervention duration, or patient age) was not feasible due to the limited number of included studies (*n* = 5). With fewer than 10 studies per subgroup, statistical power would be insufficient to detect meaningful differences. Additionally, conducting subgroup analysis with a small number of studies could lead to unreliable or biased conclusions. Publication bias was assessed using funnel plots, supplemented by Egger's and Begg's tests. A p-value of < 0.05 was considered statistically significant. All statistical analyses were conducted using Review Manager 5.4 software.

## Results

### Research, identification and selection

**Fig. 1:**
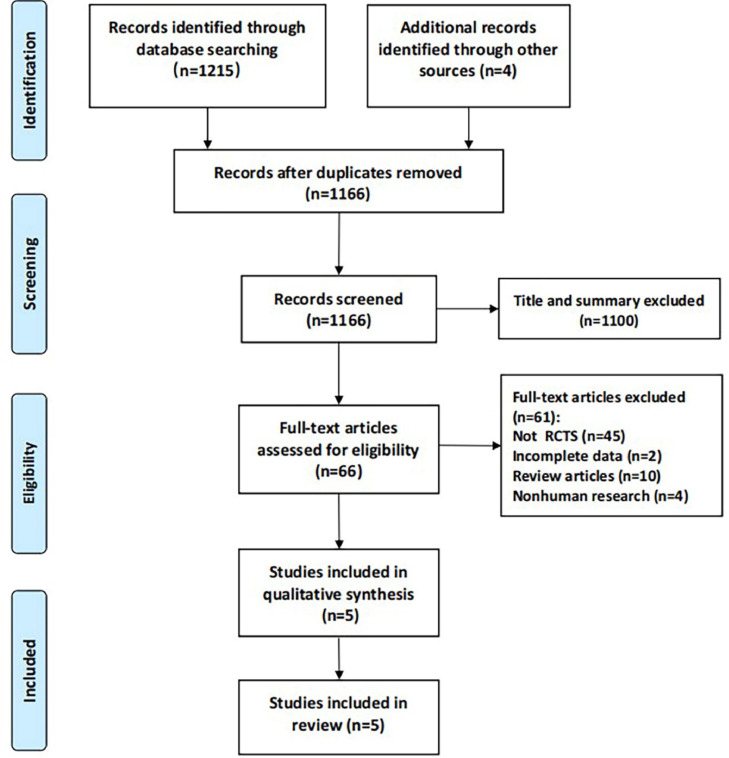
Flow chart of literature selection.

A total of 1215 articles were retrieved from the electronic databases, and an additional 4 articles were manually searched. After removing 53 duplicates, 1166 articles remained. Titles and abstracts were screened, leading to the exclusion of 1100 articles. The full texts of the remaining 66 articles were reviewed, resulting in a further 61 exclusions for the following reasons: non-RCTs (*n* = 45), incomplete data (*n* = 2), review articles (*n* = 10), and non-human research (*n* = 4). Consequently, 5 articles were included in this study (Fig. 1).

### Quality evaluation and characteristics of the included studies

**Fig. 2:**
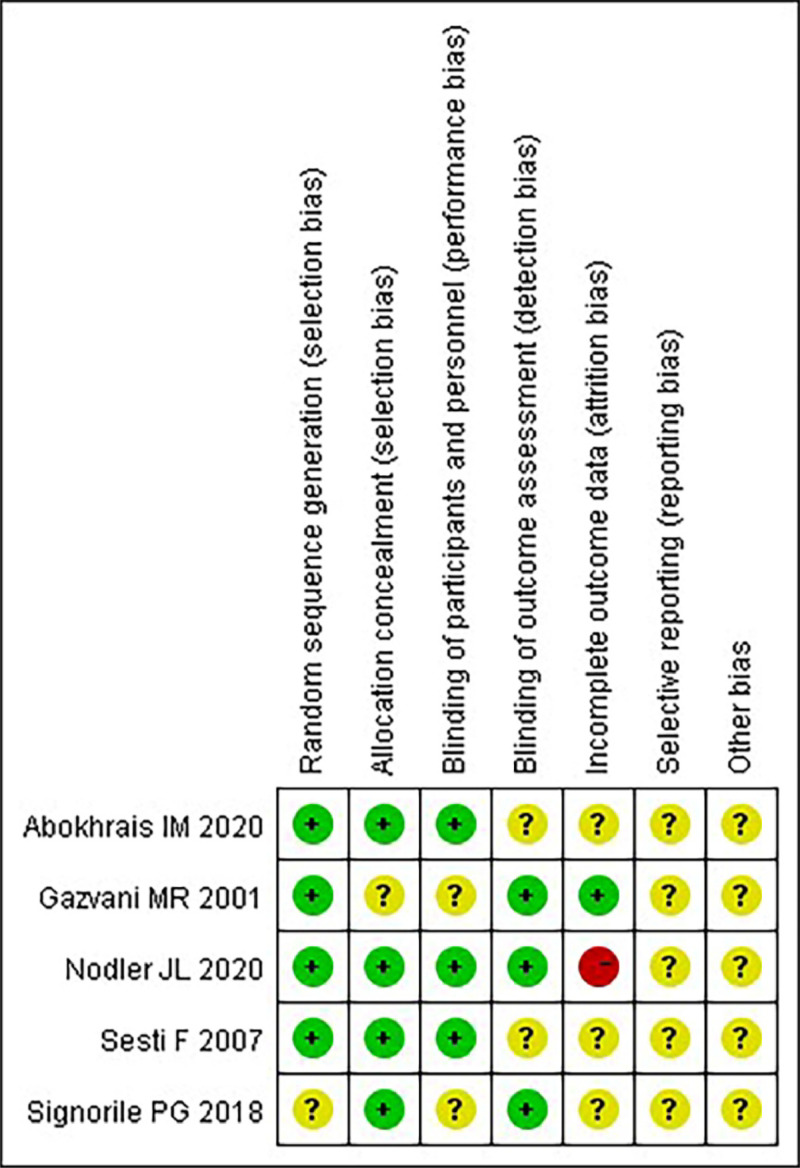
Research ROB risk bias graph.

Five RCTs were included, all categorized as medium risk (Fig. 2), involving a total of 424 patients diagnosed with endometriosis. The interventions for the control groups included oral fish oil,^24^ oral ω−3 PUFAs,^25^ dietary supplements in two studies,^26^^,^^27^ and high-dose ω−3/6 ratio fatty acids in one study.^15^ The outcomes measured included pain (three studies), improvement in sexual activity (two studies), cytokines (two studies), pain improvement interventions (two studies), results from the SF-12 survey (two studies), and catastrophic thinking (two studies). The studies were conducted globally, with one in the US, two in the UK and two in Italy. Details of the study characteristics are presented in Table 2.

**Table 2:** Characteristics of the studies included in the meta-analysis.

	Author	Country	Year	Population	Age (mean+SD)	Total	Intervention	Control	Outcome
**1**	Nodler^24^	America	2020	Endometriosis patients	C: 20.1 ± 3.5	69	Fish oil	Placebo tablet	①④⑤⑥
					T: 18.9 ± 3.1		Dose: either 1000 mg		
							Lengthof Intervention: 6 mo		
**2**	Abokhrais^25^	Britain	2020	Endometriosis patients	C: 35.43 ± 8.57	33	Omega-3-acid ethyl ester PUFA Capsules	Oliveoil capsules	①②④⑤⑥
					T: 36.08 ± 9.59		Dose: 1000 mg		
							Freq: twice a day		
							Lengthof Intervention: 8w		
**3**	Signorile^26^	Italy	2018	Endometriosis patients	C: 35.2	90	Omega 3	Placebo	③
					T: 34		Dose: 1002 mg linoleic acid, 432 mg alpha linolenic acid		
							Freq: twice a day		
							Lengthof Intervention: 3 mo		
**4**	Gazvani^15^	Britain	2001	Endometrial tissue	NR	10	High amounts of omega 3	No PUFA	③
							PUFAs		
							Dose: alpha linolenic acid-0.1, Eicosapentaenoic acid −1.3		
	Sesti^27^	Italy	2007	Endometriosis stage III–IV	C: 31.0 ± 4.0	222	Dietary therapy (omega-3 and omega-6 fatty acids)	Placebo	①②
					T: 29.0 ± 3.9		Lengthof Intervention: 6 mo		

T, Experimental group; C, Control group.

① Chronic pain; ② Sexual Activity; ③ Cytokines level; ④ Pain intervention improvement; ⑤ Short Form 12 (SF-12) questionnaire; ⑥ Catastrophic thinking.

### Result analysis

#### Pain group

**Fig. 3:**
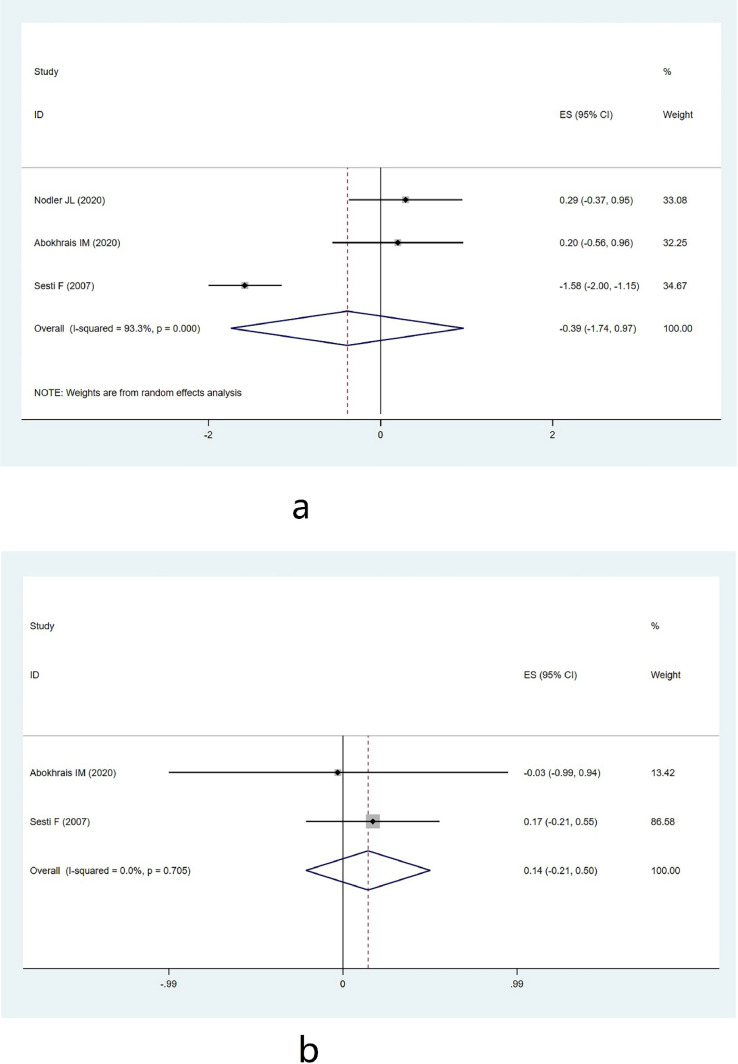
(a) Pain group forest map. (b) Sexual activity group forest map.

Three studies involving 324 participants assessed the impact of ω−3 PUFAs on endometriosis, focusing on pain as the outcome. High heterogeneity was found (χ^2^ = 30.06, *p* = 0.000, *I*^2^ = 93.3 %), and a random effects model was used for analysis. The results showed no statistically significant effect (MD = −0.387, 95 % CI −1.742–0.967, *z* = 0.56, *p* = 0.575) (Fig. 3a). This suggests that ω−3 PUFAs may not significantly reduce pain associated with endometriosis, indicating the need for further studies to explore alternative or adjunctive treatments for pain management in patients with endometriosis.

#### Sexual activity

Two studies with 255 participants examined the impact of ω−3 PUFAs on improving sexual activity in endometriosis, using sexual activity as an outcome measure. Low heterogeneity was detected (χ^2^ = 0.14, *p* = 0.705, *I*^2^ = 0.0 %), and a fixed-effects model was applied. The results indicated no statistically significant effect (MD = 0.143, 95 % CI −0.210–0.497, *z* = 0.79, *p* = 0.427) (Fig. 3b). This finding implies that ω−3 PUFAs may not significantly enhance sexual activity in patients with endometriosis, highlighting the complexity of treating sexual dysfunction in this condition.

#### Cytokine levels

**Fig. 4:**
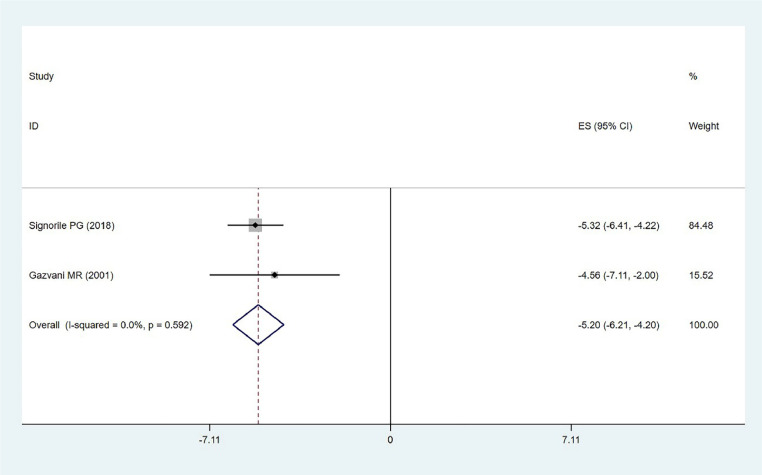
Cytokines level.

The effect of ω−3 PUFAs on cytokine levels in endometriosis was analyzed in two studies involving 100 participants, choosing cytokine levels as an outcome measure. The analysis indicated low heterogeneity (χ^2^ = 0.29, *p* = 0.592, *I*^2^ = 0.0 %), and a fixed-effects model was used. The results showed a statistically significant effect (MD = −5.20, 95 % CI −6.21–−4.20, *z* = 10.13, *p* < 0.001). The diamond-shaped plot falling to the left of the line of no effect, in the direction of the ω−3 PUFAs intervention and not crossing the line of no effect, indicates that ω−3 PUFAs reduce the impact of endometriosis compared to the control group (Fig. 4). This significant reduction in cytokine levels suggests that ω−3 PUFAs may have a potent anti-inflammatory effect in patients with endometriosis. These findings are important as they indicate a potential therapeutic benefit of ω−3 PUFAs in managing the inflammatory aspects of endometriosis, which could lead to improved patient outcomes in terms of reduced pain and other inflammatory symptoms.

#### Pain intervention improvement

**Fig. 5:**
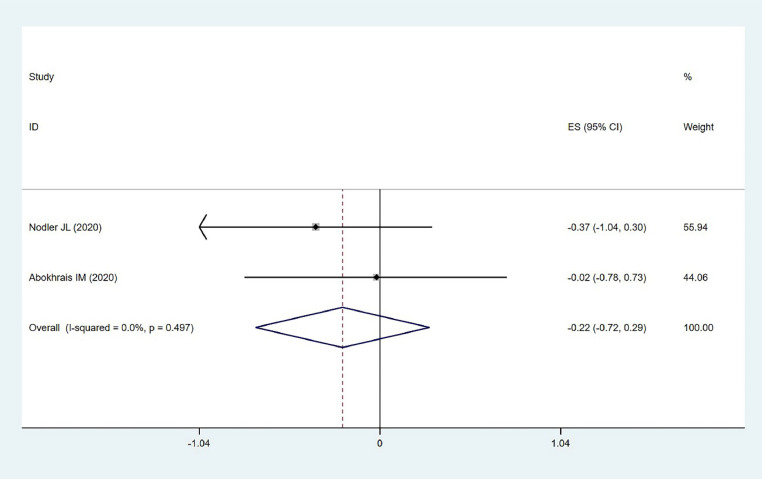
Pain intervention improvement.

Two studies involving 102 participants assessed the effect of ω−3 PUFAs on improving pain intervention in endometriosis, choosing pain intervention as an outcome measure. Low heterogeneity was found (χ^2^ = 0.46, *p* = 0.497, *I*^2^ = 0.0 %), and a fixed-effects model was used. The results showed no statistically significant effect (MD = −0.216, 95 % CI −0.717–0.285, *z* = 0.84, *p* = 0.399) (Fig. 5). This indicates that ω−3 PUFAs may not significantly enhance the effectiveness of pain interventions in endometriosis, suggesting that other therapeutic options need to be considered.

#### Twelve-item short-form health survey questionnaires

**Fig. 6:**
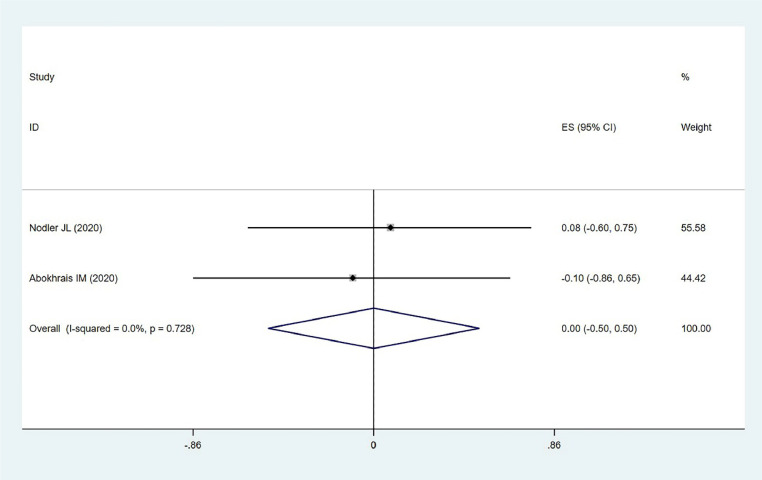
Short Form-12 (SF-12) questionnaire.

The impact of ω−3 PUFAs on endometriosis was evaluated using SF-12 surveys in two studies involving 102 individuals. Low heterogeneity was detected (χ^2^ = 0.12, *p* = 0.728, *I*^2^ = 0.0 %), and a fixed-effects model was applied. The results showed no statistically significant effect (MD = 0.001, 95 %CI −0.053–0.503, *z* = 0.00, *p* = 1.000) (Fig. 6). This suggests that ω−3 PUFAs do not have a significant impact on overall health status as measured by the SF-12 survey in patients with endometriosis, indicating that their benefits may be limited to specific inflammatory markers rather than broader health outcomes.

#### Catastrophic thinking

**Fig. 7:**
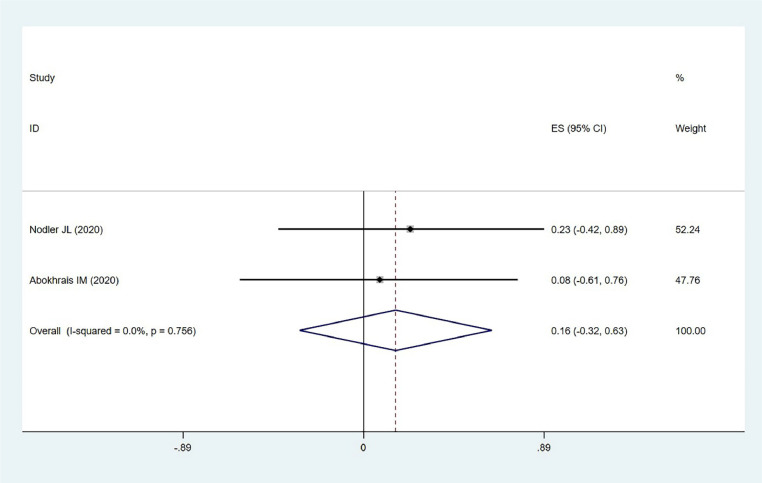
Catastrophic thinking.

Two studies involving 102 participants examined the impact of ω−3 PUFAs on catastrophic thinking in endometriosis. Low heterogeneity was observed (χ^2^ = 0.10, *p* = 0.756, *I*^2^ = 0 %), and a fixed-effects model was used. The results indicated no statistically significant effect (MD = 0.158, 95 % CI −0.315–0.632, *z* = 0.66, *p* = 0.512) (Fig. 7). This finding suggests that ω−3 PUFAs may not effectively reduce catastrophic thinking in patients with endometriosis, highlighting the need for psychological support and interventions in managing this aspect of the condition.

### Publication bias test

**Fig. 8:**
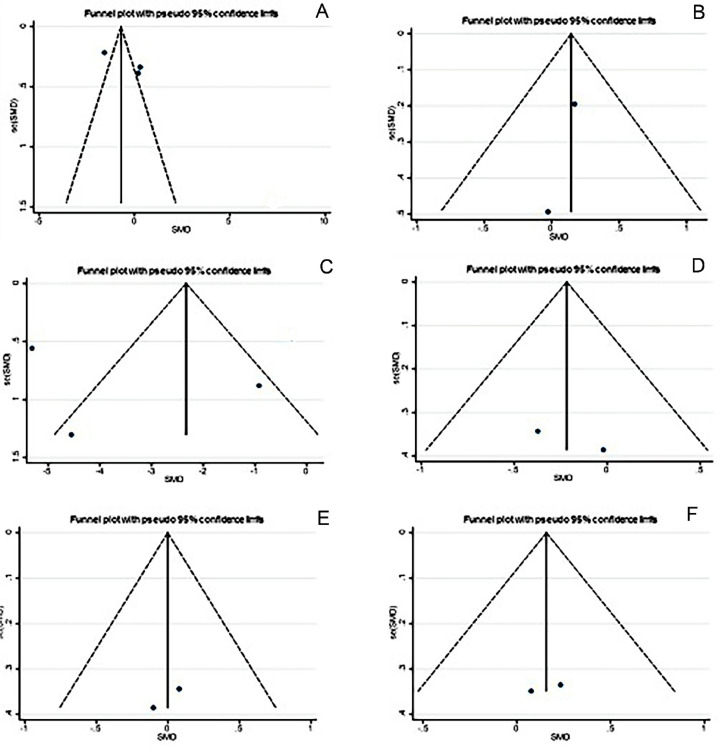
Funnel plot on publication bias. (A) Pain group, (B) Sexual activity group, (C) Cytokines level, (D) Pain intervention improvement, (E) SF-12 questionnaire, (F) Catastrophic thinking.

Separate funnel plots were constructed for all outcome indicators to assess potential publication bias. No substantial publication bias was detected^28^ (Fig. 8).

## Discussion

This meta-analysis of five RCTs (*n* = 424) found that ω−3 PUFAs did not significantly alleviate endometriosis-related symptoms, including pain, sexual dysfunction, or quality of life metrics. However, a notable reduction in pro-inflammatory cytokines (e.g., TNF-α, IL-6, IL-1) was observed, suggesting its specific anti-inflammatory effects.

The most substantial symptoms of endometriosis include pain – encompassing dysmenorrhoea, chronic pelvic pain, and deep sexual pain – and a decrease in quality of life due to pain. The present research results indicate that ω−3 PUFAs have no effect on improving these symptoms. This finding is consistent with previous research.

In contrast, conflicting results have been reported in other studies. Nodler's study also demonstrated that supplementing with fish oil for 6 months did not result in clinically or statistically significant changes in pain in adolescent girls and young women with endometriosis compared with a placebo.^24^ Arab et al. reported no substantial association between the intake of PUFAs and a reduced risk of endometriosis.^29^ Similarly, the study by Trabert et al. indicates that the intake of PUFAs is usually not associated with the risk of endometriosis.^30^ A recent double-blind, randomized and controlled trial tested ω−3 PUFAs in women with surgically confirmed endometriosis and pelvic pain; substantial improvements in pain scores were found in the vitamin D group and the placebo group. In contrast, women in the ω−3 PUFAs group showed smaller improvements.^24^

This discrepancy may be attributed to biological mechanisms that support the role of these dietary factors in influencing the risk of endometriosis. However, epidemiological data do not consistently support these hypotheses.^31^ This may be due to the lack of suitable placebos, as most of the included studies did not find placebos identical to ω−3 PUFA, resulting in the use of olive oil instead of a true placebo. Olive oil contains bioactive compounds, such as oleocanthal, which have been shown to have anti-inflammatory and pain-relieving properties. These properties can potentially influence the study outcomes by reducing pain and inflammation in the placebo group, thereby masking the true effects of ω−3 PUFAs. This could lead to negative outcomes and misinterpretation of the efficacy of ω−3 PUFAs in treating endometriosis symptoms.^32^ Additionally, effective clinical interactions and trial participation can impact pain, as well as psychological and physical health. This includes participants' experiences and pain reports, which could, in turn, affect trial results.^25^

The literature has shown that foods high in ω−6 PUFAs can increase pro-inflammatory substances.^33^ In women with endometriosis, an alteration in the ratio of ω−6 to ω−3 is associated with an increase in menstrual pain, hormonal imbalances, and autoimmune diseases.^34^ In the studies involved, the presence of both ω−6 and ω−3 PUFAs in the reagents at different doses may also lead to negative results. However, there are also contrary findings; Khanaki demonstrated that although the level of PUFA in serum total phospholipids does not appear to be a marker of endometriosis, the ratio of EPA to Arachidonic Acid (AA) in ω−3 PUFAs is a substantial factor indicating the severity of the disease.^35^ The increased level of secretory Phospholipase A2 (sPLA2) can lead to the enhanced release of AA and the synthesis of eicosanoids, thereby increasing inflammatory reactions. Khanaki et al. also found that ω−3 PUFA may increase the level of extracellular sPLA2-IIa in ectopic endometrial cells, as sPLA2-IIa could affect endometriosis through several mechanisms.^36^ Previous epidemiological studies have shown that the intake of ω−3 PUFAs and pelvic pain are negatively correlated.^37^

The research results also indicated changes in peritoneal factors, contrasting with some previous studies, which suggest that the local environment of the peritoneal fluid around endometriosis implants exhibits pre-inflammatory tissue characteristics associated with changes in immune response and cytokine production.^38^ De et al.'s study showed that ω−3 PUFAs inhibit the activation of NF-kB and reduce the formation of pro-inflammatory cytokines, such as TNF-alpha, IL-6 and IL-1.^39^ This may explain the divergence between biomarker improvements and clinical symptom outcomes. From a long-term perspective, further experimental research is necessary to elucidate the effects of ω−3 PUFAs on endometriosis in the future.

## Advantages and limitations

First, the study was registered prospectively in the International Prospective System Evaluation Register, thereby reducing the risk of reporting bias. Furthermore, the present study was conceptualized and developed by two researchers using rigorous and reliable methods. The authors meticulously extracted and repeatedly verified the data during the organization. Second, the search strategy included extensive updates to the search results, encompassing thousands of records from the literature. Finally, it reports multiple findings that were not previously combined in earlier articles.

The present research has some limitations compared with the studies upon which it builds. This analysis depends on articles cited by the original authors and results already published in systematic reviews and meta-analyses. Although the authors made every effort to control the heterogeneity of the present research when incorporating these original studies, some degree of heterogeneity between the studies is inevitable. For example, there are limitations due to the scarce research literature and small sample sizes, as well as variations in age, body mass index, study design, geographic differences, study quality, frequency of exposure, types of exposure, and the control sources of the control group, along with differing response rates between the case and control groups.

Finally, readers should interpret the results of this research with caution, as the number of studies is limited, and direct comparative evidence for some interventions is scarce. It also highlights the necessity of further expanding the relevant research.

## Conclusion

Based on the available evidence, ω−3 PUFAs may reduce the inflammatory response in patients with endometriosis, specifically by decreasing levels of pro-inflammatory cytokines, such as TNF-alpha, IL-6 and IL-1, indicating potential clinical benefits in anti-inflammatory properties. Future studies should prioritize large-scale, multi-center RCTs with standardized ω−3 PUFA dosages and placebo controls. Mechanistic research on ω−3 PUFA modulation of NF-κB and cytokine pathways in endometriosis lesions is also warranted.

## Declarations

Ethics Approval and Consent to Participate: Not applicable.

Informed Consent Statement: Not applicable.

Availability of Data and Materials: The data supporting the findings of this study are available from the first author upon reasonable request.

Consent for Publication: The manuscript has not been submitted for publication or consideration elsewhere.

## Author contributions

Liu EN interpreted the data and participated in writing the first draft. Wang QT was responsible for collecting all relevant documents, participating in writing the first draft, and in data analysis. Wang JF supervised this study. Bai YX and Zhang X were responsible for collecting all relevant documents. All authors have read and agreed to the published version of the manuscript.

## Funding

This study was supported by the 10.13039/100014717Youth Natural Science Foundation of Shanxi Province: Mechanism of laminin activated integrin receptor mediated PI3K/Akt pathway in endometriosis complicated with endometrial polyps (grant number 202403021212269) and the Shanxi Province Key National Science and 10.13039/501100012326Technology Cooperation Projects (grant num- ber 202104041101006).

## Declaration of competing interest

The authors declare no conflicts of interest.
